# A Severe Accident Caused by an Ocellate River Stingray (*Potamotrygon motoro*) in Central Brazil: How Well Do We Really Understand Stingray Venom Chemistry, Envenomation, and Therapeutics?

**DOI:** 10.3390/toxins7062272

**Published:** 2015-06-18

**Authors:** Nelson Jorge da Silva, Kalley Ricardo Clementino Ferreira, Raimundo Nonato Leite Pinto, Steven Douglas Aird

**Affiliations:** 1 Programa de Pós-Graduação em Ciências Ambientais e Saúde, Pontifícia Universidade Católica de Goiás, Rua 232 nº128 3º andar-Área V. Setor Universitário, CEP 74605-140 Goiânia, Goiás, Brazil; E-Mail: nelson.jorge.silvajr@gmail.com; 2 Faculdade de Ciências Médicas do Instituto de Pesquisa e Ensino Médico – IPEMED, Rua Dr. Nogueira Martins nº 706, Saúde, CEP 04143-020 São Paulo, São Paulo, Brazil; E-Mail: kalley.ferreira@me.com; 3 Departamento de Medicina, Pontifícia Universidade Católica de Goiás, Avenida Universitária, 1069-Setor Universitário, CEP 74605-010 Goiânia, Goiás, Brazil; E-Mail: raimundo58@gmail.com; 4 Hospital de Doenças Tropicais Dr. Anuar Auad. Rua SC-1 nº 299, Parque Santa Cruz, CEP 74860-270 Goiânia, Goiás, Brazil; 5 Evolution and Ecology Unit, Communications and Public Relations Division, Okinawa Institute of Science and Technology Graduate University, 1919-1 Tancha, Onna-son, Kunigami-gun, Okinawa 904-0495, Japan

**Keywords:** stingray envenomation, *Potamotrygon motoro*, treatment, antibiotics, venom chemistry, pharmacology

## Abstract

Freshwater stingrays cause many serious human injuries, but identification of the offending species is uncommon. The present case involved a large freshwater stingray, *Potamotrygon motoro* (Chondrichthyes: Potamotrygonidae), in the Araguaia River in Tocantins, Brazil. Appropriate first aid was administered within ~15 min, except that an ice pack was applied. Analgesics provided no pain relief, although hot compresses did. Ciprofloxacin therapy commenced after ~18 h and continued seven days. Then antibiotic was suspended; however, after two more days and additional tests, cephalosporin therapy was initiated, and proved successful. Pain worsened despite increasingly powerful analgesics, until debridement of the wound was performed after one month. The wound finally closed ~70 days after the accident, but the patient continued to have problems wearing shoes even eight months later. Chemistry and pharmacology of *Potamotrygon* venom and mucus, and clinical management of freshwater stingray envenomations are reviewed in light of the present case. Bacterial infections of stingray puncture wounds may account for more long-term morbidity than stingray venom. Simultaneous prophylactic use of multiple antibiotics is recommended for all but the most superficial stingray wounds. Distinguishing relative contributions of venom, mucus, and bacteria will require careful genomic and transcriptomic investigations of stingray tissues and contaminating bacteria.

## 1. Introduction

Freshwater stingrays are elasmobranchs belonging to the monophyletic Family Potamotrygonidae, Garman, 1877. Descendants of Pacific and Caribbean marine stingrays [1], potamotrygonids exhibit considerable diversity in South America [2,3,4,5,6,7,8]. They are currently classified into four genera: *Paratrygon* (Duméril, 1865), *Potamotrygon* (Garman, 1877), *Plesiotrygon* (Rosa, Castello and Thorson, 1987), and *Heliotrygon* [9]. The Genus *Potamotrygon* is the most diverse, with more than 25 species identified to date [6,9,10,11,12,13]. Potamotrygonid stingrays occur in most river basins that drain into the Atlantic Ocean, including the Amazon-Orinoco, the Paraguay-Paraná, the Uruguay, and the Parnaíba River basin in northeastern Brazil [3,6,14,15,16,17,18].

Diurnally, stingrays partially bury themselves in sand or mud in shallow portions of rivers and lakes, where they feed mainly on small invertebrates and fish [10,19,20]. Stingrays possess one to three barbed stingers in the mid-distal region of the tail. These are covered with secretory cells that produce various proteins having nociceptive, inflammatory, and necrotic actions [14]. In addition, stingrays, like other fish, are covered with mucus. Even though the mucus contains elements of the non-specific immune system, it also harbors bacteria of many types. The stingers, with their associated secretory cells and mucus, constitute the only physical defensive weapon of these fishes [6,21,22,23,24,25,26,27,28]. They have no offensive or prey-capture function.

South American freshwater stingrays cause a large number of serious human injuries. These are common on the feet, ankles, and distal parts of the leg when people accidentally step on them, but because captured rays tend to thrash violently, fishermen also commonly suffer injuries to the hands and arms [6,7,21,23]. In central Brazil, the Tocantins and Araguaia River basins (subsystems of the Amazon-Orinoco basin) are heavily used for recreational activities, especially from June to August. Being the dry season, low water levels leave exposed beaches that attract tourists, and during this season stingray accidents are most common.

In stingray envenomations, the mechanical injury itself is excruciatingly painful and usually causes considerable damage, owing to the retrorse serrations of the stingers, which sometimes cause trauma to major nerves and blood vessels [23,29,30,31,32,33]. The physical damage is exacerbated by the action of substances present in the mucus and the secretory epithelium covering the stingers. Secondary bacterial infections involving gram-negative species are common [34,35]. Serious morbidity and mortality are especially common in rural areas where medical attention is not readily available and patients do not seek treatment until symptoms become severe. Prompt and intensive cleansing of the wound, tetanus prophylaxis, and appropriate antibiotics are crucially important [36,37,38]. Domingos *et al.* [34] reported that antibiotic resistance of bacteria associated with stingray mucus is common, especially to ampicillin, amoxycillin/clavulanic acid, and cephalotin; 23% of their bacterial isolates were resistant to all but one of the sixteen antibiotics they tested. In addition to extreme nociception, stingray envenomations often involve edema, inflammation, and myotoxicity, while hemorrhagic and hemolytic activities are absent [24]. Severe envenomations involving delayed medical treatment or clinical mismanagement can result in amputation or death [38,39].

*Potamotrygon motoro* (Müller and Henle, 1841 [18]), the ocellate river stingray, is one of the most common stingray species in the Tocantins-Araguaia river basin, and has one of the broadest geographic distributions of any species in the genus. Despite the number of epidemiological studies of stingray envenomations, positive identification of the stingray species involved is quite rare [38]. In this paper, we report a severe human accident involving an ocellate river stingray in central Brazil and we discuss implications for public health strategies related to freshwater stingray envenomations. What makes this case unusual is that the victim, one of the co-authors of this report, was raised in the region where the accident occurred. He is an ardent fisherman familiar with fish of the local rivers. When shown photographs of various rays, marine and freshwater, Brazilian and foreign, he unhesitatingly identified the offending species. Moreover, he is also a physician. Accordingly, the resulting case report is the most detailed of which we are aware.

## 2. Case Report

**Figure 1:**
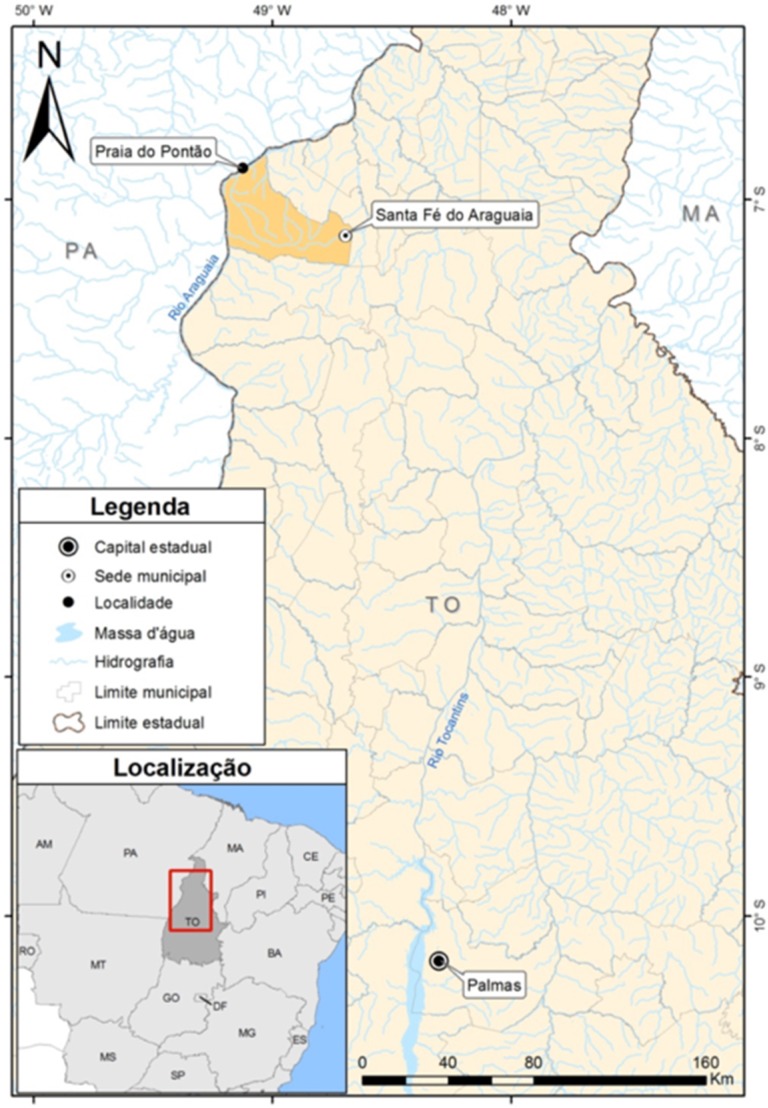
Map showing the location of the stingray accident in northwestern Tocantins state with landmarks mentioned in the text. Drawing: Sérgio Henrique de Moura Nogueira (2015).

**Figure 2:**
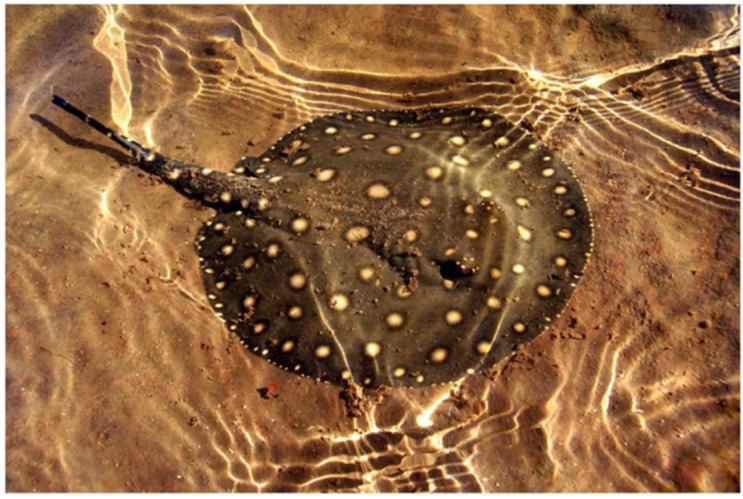
An adult ocellate river stingray (*Potamotrygon motoro*) from the Araguaia-Tocantins basin. Photo: Itamar Júnior Tonial (2013).

On 20 July 2014, at approximately 12:30 pm, a 41-year-old Caucasian male was stung by a freshwater stingray along a beach of the Araguaia River, known as Praia do Pontão (municipality of Santa Fé do Araguaia) in the northwestern region of the state of Tocantins, Brazil (Figure 1). While bathing in shallow water at the beach, the subject felt something under the sand, but before he could react, he was stung on the medial face of the left foot. The victim, despite the stress of the situation, was able to spot and carefully observe the stingray. It was an adult ocellate river stingray (*Potamotrygon motoro*) approximately 60 cm in width (across the body disk) (Figure 2).

**Figure 3:**
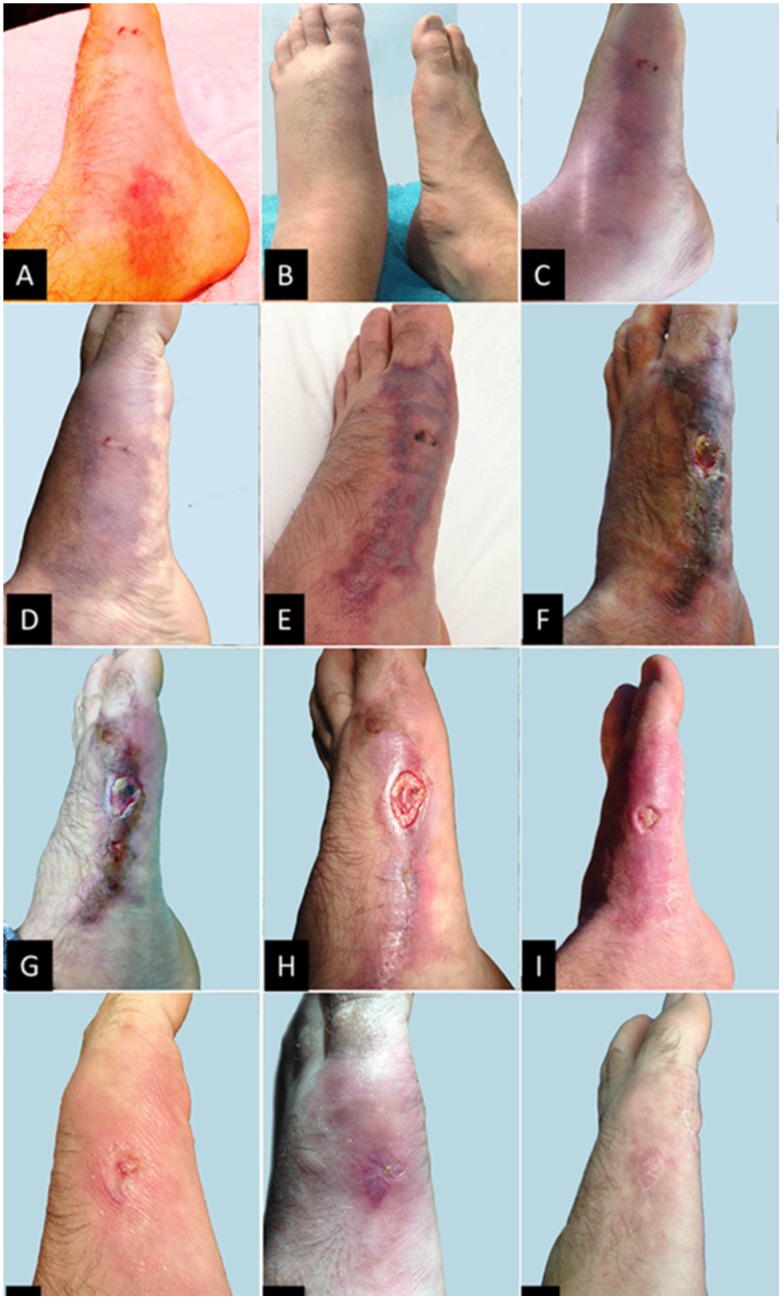
Evolution of the accident caused by an ocellate river stingray (*Potamotrygon motoro*) from the first day to complete healing of the wound: **A**. Evident edema with vasculitis and hyperemia caused by the trauma of sting penetration with laceration of tissues, and by the action of proteolytic enzymes that damage cells and liberate fluid and cellular debris into the interstices; **B**. Worsening of edema 24 h after the accident; **C**. Evident ecchymosis and prevailing edema; **D**. Worsening of ecchymosis; **E**. Ecchymosis with evident initial local necrosis; **F**. Dry necrosis of the affected area with evident dead tissue; **G**. Replacement of necrotic tissues with granulation tissue, although with areas of dead tissue; **H**. Total replacement of necrotic area with granulation tissue and evident recovery; **I**. Accelerated granulation of the affected area with only the focal trauma remaining open; **J**. to **L**. Total recovery of tissues and regeneration of the traumatized area.

The accident resulted in a deep puncture wound with copious bleeding and excruciating pain that quickly spread to the distal part of the thigh. Reaching a local first aid station 10 min after the accident, the wound was cleaned with Povidone iodine and the patient received 3 mL of 2% Xylocaine subcutaneously, and 1.5 g Paracetamol (acetaminophen), a non-steroidal, anti-inflammatory (Diclofenac Potassium, 75 mg), and an ampyrone sulfonate analgesic, antispasmodic, and antipyretic Dipyrone (1 g) orally. At that time, an ice compress was applied and the victim was transported by boat to the nearest village (40 min) and then by car (2 h) to the town of Santa Fé do Araguaia (Figure 1). Admitted to a local hospital, the victim received an ampoule of analgesic (Tramadol Hydrochloride, 100 mg) and a synthetic opioid analgesic (Meperidine, 100 mg) *i.v.*; however, these did not reduce the pain. By this time, edema and erythema were strongly evident. The site of the wound was extremely sensitive to touch, and the pain had radiated to the upper left thigh. Inguinal lymph nodes on the left side were visible enlarged with the first signs of peripheral vasculitis (Figure 3A). Only at this point was the patient informed that the ice compress was inappropriate. It was replaced with hot compresses, which caused the local pain to subside within a 3 h period.

On 21 July, the patient was transported by car to Palmas, the capital city of Tocantins, an 11 h trip (Figure 1). During this journey, clinical symptoms worsened considerably, with increased edema and pain (Figure 3B). In a hospital environment, the wound was again cleaned with Povidone and the patient received another 3-mL dose of 2% Xylocaine subcutaneously. Antibiotic therapy was initiated approximately 18 h after the injury and analgesic and anti-inflammatory medications were changed. The patient received the antibiotic (Ciprofloxacin, 500 mg) plus a non-steroidal, anti-inflammatory (Ketoprofen, 50 mg), and Tramadol Hydrochloride (50 mg), each three times a day. He remained at Palmas 48 h before being transferred to São Paulo, where he resides. During this period the edema began to subside, while the intense local and radiating pain persisted (Figure 3C).

On 24 July, the patient was transferred to São Paulo by air. Once again in a hospital environment, the patient was attended to by a vascular surgeon, who requested a blood count and imaging exams (x-ray, tomography and echo-doppler). The results revealed a peripheral vascular lesion and evident leukocytosis (12,500 leukocytes/mL; 92% neutrophils; elevated Protein C). Medication started on 21 July was continued. However, the pain was not alleviated. The patient started to walk using crutches. On 27 July, with no fever or signs of local infection, antibiotics were suspended, (Figure 3D). However, on 29 July, after repeating all laboratory tests, antibiotic therapy was reinitiated, this time with a beta-lactam antibiotic (Cephalosporin, 2 g, *i.v.*) once a day, for seven days (until 4 August) with the persistence of pain and worsening of the local lesion (Figure 3E).

The period from 5–20 August comprised the most serious phase of the local lesion with vasculitis and tissue necrosis. On 5 August, the patient received Tramadol Hydrochloride (3 × 50 mg/day), 200 mg of a medication for neuropathic pain (Carbamazepine, 3 × 200 mg/day), and thiamine (1 × 300 mg/ day) on suspicion of peripheral neuropathy. With still worsening pain, this protocol was changed to include Ketoprofen (1 × 150 mg/ day), Oxycodone (2 × 10 mg/day), and Clexane (low MW heparin) (1 injection *s.c.* × 60 µg) every 24 h. The latter was given in consequence of the patient’s vasculitis. This regimen was maintained until 17 August (Figure 3F). The patient was also given Zolpidem (10 mg/day) for two months to treat insomnia. Interestingly, during this period the patient began to experience significant symptoms of anxiety. He was given escytalopram (10 mg/day) and cognitive behavioral therapy was initiated. Therapy continued until the end of November, but escytalopram was continued until March, 2015.

On 19 August, debridement of the wound was performed, which subsequently decreased the pressure and pain (by 21 August). Pain medications were changed accordingly. At this point the patient received only Ibuprofen (600 mg) and Paracetamol (750 mg) as needed (Figure 3G,H). On 21 August, the wound started to granulate along the margin. Bandages with SAF-Gel and Kollagenase topical cream (0.6 U/g) were applied every other day. After five days, the peripheral vasculitis persisted without any advance in granulation. Significant local pain and functional incapacitation of the left leg required the continued use of crutches. The left foot became increasingly painful when touched (Figure 3I).

Beginning on 9 September, bandages with topical Phenytoin cream (4%) were changed once a day. The healing process (cicatrization) accelerated significantly and the wound closed completely between 20 and 30 September, when the patient started to move without the aid of crutches (Figure 3J,K). The region around the wound was still sensitive to touch as of 1 March 2015, and the patient continued to have problems wearing closed shoes (Figure 3L).

## 3. Discussion

There have been relatively few biochemical characterizations of freshwater stingray venoms. Regarding potamotrygonid venoms, Rodrigues (p. 683) [40] declared that, “The chemical nature of the active principle is unknown, and our knowledge gathered from previous research is of dubious significance”. More than 40 years later, it is probably still safe to say that we know more about what these venoms are not, than what they are. Marine stingray venoms have been somewhat better characterized than those of their freshwater cousins [21,41], but until very recently that was not saying much [24].

Understandably, stingray venoms share more attributes with fish venoms generally than with those of other vertebrates or invertebrates. However, they are difficult to characterize because they are not produced in a gland with a central lumen, but rather as a layer of secretory cells on external spines. Additionally, stingray venoms are mucus-rich, chemically unstable, and are adversely affected by heat and pH. They lose activity with increasing storage time, freezing and thawing, and even lyophilization [42].

How similar potamotrygonid venoms are to those of marine rays is also unclear. The most thorough characterization of any ray venom to date is a transcriptomic and proteomic study of *Neotrygon kuhlii* venom by Baumann *et al.* [41]. Venom proteins with identified functions that are probably pertinent to envenomation include galectin, two cystatins, and peroxiredoxin-6. They did not find orpotrin or porflan (*Potamotrygon orbignyi*) [43,44], although they did manage to isolate a hyaluronidase gene; however, it was not present in the venom proteome, so they concluded that it was not actually a venom protein. They did not indicate whether it was of vertebrate or bacterial origin. Accidents caused by freshwater stingrays also tend to be more serious than those caused by marine rays [23]. Taken together, these reports suggest that characterizations of marine ray venoms may offer limited insights into the chemistry and pharmacology of freshwater ray venoms.

### 3.1. Characterizations of Potamotrygon Venoms

Rodrigues [40] isolated a compound from *Potamotrygon motoro* stings that contracted guinea pig ileum and produced a lethal vasodilation in rats. He concluded that the active constituent is non-proteinaceous, non-lipidic, water-insoluble, thermostable, and parasympathomimetic. Centrifuging the solution caused a significant reduction in activity of the supernatant, suggesting that the active principle was suspended, rather than dissolved.

Specialized cells of the *Potamotrygon* stinger epithelium stain well with bromophenol blue, indicating a high-protein content [25], but it is not clear that this material comprises active constituents of the venom. Given that the “venom” collected actually consists of cellular contents, much of the protein demonstrated in protein assays [45] may be irrelevant to biological activity assays performed. While Halstead [21] opined that venom-producing cells would be of a holocrine type, Pedroso *et al.* [25] found no evidence that these cells are capable of releasing material to the environment. They postulated instead that when the stinger enters the victim’s tissues, mechanical abrasion probably shears and ruptures the venom-producing cells into the victim’s tissues.

Magalhães detected 5’-nucleotidase, phospholipase, acid phosphatase, hyaluronidase, caseinolytic, gelatinolytic and elastinolytic activities in *P. motoro* venom from animals collected in the Crixás-Açú River (Goiás, Brasil) [46]. Haddad *et al.* [23] similarly reported hyaluronidase, caseinolytic, gelatinolytic and elastinolytic activities in *P. falkneri* venom. The latter authors [23] noted that *P. falkneri* venom has a major component with a mass of ~12 kDa. SDS PAGE showed seven components between 80–200 kDa. Components with masses of 80+ and 100 kDa displayed gelatinolytic and caseinolytic activities, respectively. Hyaluronidase activity was also present and was attributed to a protein of ~84 kDa [23,24,38].

Magalhães *et al.* [26] characterized the hyaluronidase isolated from the venom of *P. motoro*, with an estimated molecular weight of 79 kDa. Interestingly, they reported that 97% of the hyaluronidase activity was lost during gel filtration. If bacteria account for much of the hyaluronidase activity, they probably would have been filtered out by the gel. The enzyme was thermolabile, losing 70% of its activity when incubated for 30 min at its thermal optimum of 40 °C [26].

*Potamotrygon* venoms are well known to be nociceptive [45], but the compounds responsible are unknown. Heating of *P. orbignyi* and *P. scobina* venoms to 56 °C reduced nociceptive activity by only 32%–34% and edematogenic activity by 16%–25% of their respective levels at 37 °C [45].

Conceição *et al.* [43] reported the presence of a vasoconstrictive nonapeptide (orpotrin) from the venom of *Potamotrygon orbignyi*. It transiently reduced the diameter of mouse cremaster muscle arterioles by about 65%, with peak activity being seen approximately 20 min after injection. Later, Conceição *et al.* [44] sequenced and characterized an 18-residue pro-inflammatory peptide from venom of *P. orbignyi*. Named Porflan, it showed no similarity to any known peptide. Porflan and synthetic analogs did not affect arteriolar diameter or vascular permeability, but promoted leucocyte recruitment and adhesion in mouse microcirculation. More recently, Conceição *et al.* [47] also reported the presence of an antimicrobial protein (~16 kDa) with strong homology to the β-chain of hemoglobin. It had broad-spectrum activity against gram-negative and gram-positive bacteria and yeast, and it also exhibited minor pro-inflammatory activity. Kirchhoff *et al.* [48] found that adult *P. leopoldi* venoms have ~5-fold lower protein concentrations than juvenile venoms and that the adult venoms exhibited 10-fold lower cytotoxicity. Since stingray venoms are employed solely for self-defense, they attributed this to reduced predation pressure on adults.

### 3.2. Components Responsible for the Pharmacology of Stingray Venom and Mucus

Mucus from external surfaces of bony fishes contains a number of immune components such as lysozyme, immunoglobulin, complement, carbonic anhydrase, lectins, crinotoxins, calmodulin, C-reactive protein, proteolytic enzymes, and antimicrobial peptides (Alexander & Ingram, 1992). These probably function primarily in immune defense against bacteria and fungi. Mucus that covers the external surfaces of stingrays may be similar in composition and function. If so, any functions that the mucus serves in envenomation may be fortuitous.

There have been few attempts to characterize the pharmacology of stingray mucus. The most thorough study to date is that of Monteiro-dos-Santos *et al.* [49], who reported that both venom and mucus from the sting, and mucus from the skin of *P. henlei*, augmented vascular permeability and promoted nociception in mice, in essentially the same way as venoms and mucus of *P. orbignyi* and *P. scobina*. In both assays, the venom-mucus mixture from the sting was slightly more potent than mucus alone from the body of the ray. However, stingray mucus is well populated with bacteria and probably with some fungi as well [34,35,36,50,51,52]. Monteiro-dos-Santos *et al.* [49] do not appear to have filtered their solutions so as to remove bacteria, but we are unaware of any studies that have. This raises some interesting and clinically important questions, since many activities ascribed to stingray venom are extracellular enzymatic activities associated with bacterial species present in South American rivers and/or in stingray mucus [53].

- What biochemical components are produced by stingrays specifically to serve as toxins?
- To what extent do cytoplasmic components of stingray venom-producing cells contribute, if at all, to early symptoms of stingray envenomation?
- Does stingray mucus itself possess pharmacological activity relevant to envenomation?
- Why is the pain caused by freshwater stingray envenomations so intractable and persistent?
- What non-mucoid components of mucus, if any, are produced by bacteria in the mucus, rather than by the stingrays themselves?
- To what degree do bacteria in the mucus and in the water contribute to the sequelae of stingray envenomation?

It will not be possible to answer these questions conclusively without careful transcriptomic and genomic studies of both stingray venom-producing cells and bacteria present in stingray mucus. Clinically, the last question may be the most important, as the current case report suggests.

### 3.3. Clinical Treatment of the Patient

Proper treatment for stingray envenomations remains poorly understood and somewhat controversial within the Brazilian medical community. There are even anecdotal reports of anti-*Bothrops* antivenin being administered in some cases due to the pain and inflammation presented by stingray victims. This is unquestionably ineffective. The recommended therapeutic approach employs analgesics and anti-inflammatory drugs, immersion of the injured body part in hot water for pain relief, and antibiotics to prevent bacterial septicemia, gangrene, and tetanus [23,27,28,37]. In the present case, different treatment regimens were employed at different stages of the case, some more effective than others.

Ice compresses were applied for approximately the first 3 h. This treatment has been employed in some other cases [54,55], but as early as 1956, Russell and Lewis [56] concluded that heating the wound helped to control pain. Russell *et al.* [57] reported that packing the wound in ice actually intensified it. They concluded that venom components were thermolabile and that heating the injured appendage was beneficial therapeutically, a finding supported by others, with the caveat that it be done as soon as possible [37,38,58,59]. In this case also, upon arrival at the hospital in Santa Fé do Araguaia, hot compresses caused the pain to gradually subside. Magalhães *et al.* [45] reported partial inactivation of nociceptive and edematogenic activity of two *Potamotrygon* venoms at 56 °C, and Haddad Jr. *et al.* [38] recommended immersion in 60 °C water! This is too hot, a concern also expressed by Meyer [59], who recommended that the bath be only as hot as the patient can stand. However, the reduction in activity of venom components at physiologically tolerable temperatures would be significantly less than that reported by Magalhães *et al.* [45]. Meyer acknowledged the prompt relief from pain afforded by immersion in hot water but suggested that inhibition of venom components may be less significant than the direct anti-nociceptive effects of heat. This could be true; however, since Magalhães *et al.* [45] heated the venom itself, rather than affected tissue, there is some direct effect as well.

The prompt onset and the extreme persistence of intractable pain induced by stingray venoms are difficult to explain. While these venoms are well known to be nociceptive, the nociceptive compounds have not been identified, nor have possible pharmacological mechanisms been proposed. While peptidyl toxins or chemical mediators of inflammation, such as histamine, could induce pain almost immediately, they would probably be cleared in due course and the pain they induced would likely not persist for extended periods. Chemistry and pharmacology of the stingray mucus itself are unknown, but again, it seems likely that the mucus would soon be degraded by various hydrolases. While purely hypothetical at this point, perhaps the most reasonable explanation would be some form of physically or chemically induced nerve damage.

Within two hours of the envenomation, the patient manifested edema, erythema, and the first signs of vasculitis. It would not be surprising if *P. motoro* venom contains a homolog to the pro-inflammatory peptide reported from *P. orbignyi* venom by Conceição *et al.* [44], but the mechanical damage and the introduced stingray mucus, toxins, cellular debris, and bacteria would also have provoked the release of chemical mediators of inflammation. It is therefore difficult to assess how much of a contribution such a venom peptide might make to the overall picture.

We are unaware of any other reports of insomnia or anxiety associated with stingray envenomation. If purely psychological in origin, such symptoms would logically be expected immediately after the event. However, in this case, onset was almost a full month after envenomation. This suggests a possible biochemical basis for such effects, but there are no clues in the envenomation literature regarding possible causes.

Significantly, in the present case, no antibiotics were administered for approximately 18 h after the injury, despite the well-documented occurrence of pathogenic bacteria and fungi in stingray venom and mucus, and in aquatic environments generally [34]. Given that secondary bacterial infections involving gram-negative species are common, this may have been a serious oversight. Such infections have been attributed to *Aeromonas hydrophila*, *Aeromonas sobria*, *Pseudomonas aeruginosa*, *P. putida*, *Citrobacter freundii*, *Escherichia coli*, *Enterobacter cloacae*, *Photobacterium damsela*, and *Vibrio alginolyticus* [34,35]. *Clostridium perfringens*, *C. tetani*, *Pasteurella* sp., Group A *Streptococcus* and *Staphylococcus* sp. have also been reported [29,36]. These infections often involve tissue necrosis, gangrene, tetanus, and septic shock, with extreme cases leading to death. Most of the gram-negative species are found in both river water and mucus of the barb epithelium; however, some bacteria have been detected only in mucus (*Pseudomonas aeruginosa*, *Acinetobacter sp.*, *Klebsiella pneumoniae*, and some gram-positives) [34].

The first antibiotic employed was ciprofloxacin. *Aeromonas hydrophila*, one of the primary species responsible for bacterial infections of freshwater stingray wounds, is normally susceptible to ciprofloxacin; however, resistant strains have been documented in leeches [60] and in wastewater [61]. Resistance has also been reported in other bacteria common to stingray mucus, including *Pseudomonas aeruginosa* [62], *Staphylococcus aureus* [62,63], *Klebsiella pneumoniae* and *Escherichia coli* [64], *Acinetobacter sp.* [65], and *Clostridium perfringens* [66]. Ciprofloxacin and other medications commenced in Palmas were continued in São Paulo for a total of seven days. At that time, the patient manifested no fever and showed no signs of infection, so ciprofloxacin was discontinued. However, two days later, despite the lack of fever, additional lab tests showed elevated C-reactive protein (an indicator of inflammation that activates the complement system) and white blood cell counts. Accordingly, antibiotic therapy was resumed for another seven days, this time with cephalosporin.

The failure of ciprofloxacin therapy is not surprising, given the variety of bacterial species likely to be injected in a typical envenomation, and given the finding of Domingos *et al.* [34] that 23% of all bacterial isolates from stingray mucus and river water were resistant to all but one of 16 antibiotics tested. As an added caution, ciprofloxacin and other fluoroquinolones can cause peripheral neuropathy that may be irreversible, such as weakness, burning pain, tingling, or numbness [67,68,69]. In rare cases, such symptoms, should they occur, might be overlooked or misattributed to the envenomation itself. While cephalosporin therapy ultimately proved successful, resistance to this antibiotic is also well known in various bacterial species pertinent to *Potamotrygon* envenomations: *Citrobacter freundii* [70,71,72], *Pseudomonas aeruginosa*, *Aeromonas hydrophila*, *Enterobacter*, *Acinetobacter sp.*, *Klebsiella pneumoniae* [72,73,74]. Given the practice of using the same antibiotic classes for both human and veterinary applications and indiscriminate uses of antibiotics, it is virtually certain that antibiotic resistance will become increasingly problematic [72].

When the patient in the present case arrived in São Paulo, skin was starting to slough near the margins of the wound, a condition that has also been reported in *Potamotrygon falkneri* envenomations [28]. Schechter [75] reported that the basement membrane of the dermal-epidermal junction is highly susceptible to degradation by neutral proteases released by inflammatory cells. Elastase and Type IV collagenase were the most efficient at destroying the basement membrane, but trypsin- and chymotrypsin-like proteases were also capable of doing this. Gelatinases (metalloproteases) as well as collagenase have been implicated in epidermal detachment [76].

Neither of these activities has been directly linked to stingray venom; however, if not produced by mast cells or other inflammatory cells in the wound, such activity certainly could have been bacterial in origin. Collagenase activity has been reported in *Streptococcus pyogenes* and *S. mutans* [77,78], various *Clostridium* species [79,80,81,82], *Pseudomonas aeruginosa* [83,84], and *Vibrio alginolyticus* [85,86,87,88,89], being especially prominent in the latter two species. Gelatinase activity has also been reported in bacterial species present in stingray envenomations: *Aeromonas hydrophila*, *Aeromonas sobria*, *Pseudomonas aeruginosa* [34], and *Vibrio alginolyticus* [89], and also in *Streptococcus pyogenes* [77].

## 4. Conclusions and Future Directions

Given that stingray envenomations usually involve puncture wounds in aquatic environments, except in cases involving only superficial lacerations, we think it advisable to use prophylactic antibiotic treatment. Moreover, in view of the diversity of bacteria likely to be involved in a single accident and the frequency of antibiotic resistance among bacterial species present in stingray mucus and in tropical aquatic habitats, it would be wise to administer combinations of broad-spectrum antibiotics, including those effective against species of *Clostridium*.

Future studies undertaken to better characterize stingray venoms need to control for bacterial contamination of venom and mucus samples. In particular, pharmacological activity of the mucus itself needs to be carefully investigated. Even proteins isolated from mucus and venom cannot simply be assumed to originate with the stingrays themselves. Because they may be secreted bacterial proteins, the biological origins of proteins in stingray mucus need to be identified. This will necessitate genomic and transcriptomic studies of both stingray tissues and bacteria.
